# Consecutive case series of Takotsubo cardiomyopathy: a disease potentially triggered by the Great East Japan Earthquake

**DOI:** 10.1186/cc10793

**Published:** 2012-03-20

**Authors:** T Suzuki, S Sakai, T Abe

**Affiliations:** 1 Mito Kyodo General Hospital, University of Tsukuba, Mito City, Japan

## Introduction

Takotsubo cardiomyopathy (TC) is a rare disease that mimics ST elevated myocardial infarction (STEMI). TC is known to involve psychic or physical stressors such as a devastating disaster, but those clinical features have been not fully investigated. As Ibaraki prefecture suffered from the Great East Japan Earthquake, we tried to clarify the characteristics of TC and investigate whether the Great East Japan Earthquake increased the occurrence of TC or not.

## Methods

Eleven consecutive patients with TC (five men, six women) were enrolled between October 2009 and October 2011 in this study. Patients were diagnosed by echocardiography, left ventriculography, or nuclear scintigraphy. Absence of significant coronary stenosis was confirmed by coronary angiography or coronary computed-tomography angiography. Clinical characteristics (age, season, coronary risk factors, the condition that preceded onset as possible triggering factors and so on), laboratory data (troponin T, creatinine kinase, and so on) and data of electrocardiography (ECG) were obtained from reviewing medical records.

## Results

The number of cases of TC after the earthquake was five for 7 months and that of before is six for 17 months. The occurrence rate of TC seemed to increase after the earthquake. Reviewing all of our cases, 45.5% (*n *= 5/11) of patients have TC in the autumn, 72.7% (*n *= 8/11) of patients suffered from a physical stressor, and 27.3% (*n *= 3/11) of patients a psychic stressor. No obvious stressor was found in only one patient. The patients complained of chest pain or dyspnea (54.5% each). The rate of coronary risk factors were; family history, 10% (*n *= 1/10); smoking, 60% (*n *= 6/11); diabetes, 57.1% (*n *= 4/7); hypertension, 63.6% (*n *= 7/11); dyslipidemia, 44.4% (*n *= 4/9); and obesity, 22.2% (*n *= 2/9). Laboratory data showed that elevated troponin T was observed in 60% (*n *= 6/10), high CK and CK-MB were 45.5% (*n *= 5/11) and 100% (*n *= 9/9), respectively. ECG findings of all of the patients; ST elevation was observed in precordial leads of V2 to V4 (27.3%, 54.5% and 27.3%, respectively) and ST depression was in V5 (36.4%). Reversed r progression was observed in 18.2%, poor r progression was 27.3%, abnormal Q was 18.2%, long QT interval was 72.7% and negative T was 63.6% of TC patients.

## Conclusion

Although TC seems to mimic anterior STEMI, limb leads did not tend to show ST change in ECG in our cases. The Great East Japan Earthquake could increase patients with TC until the tremendous damage caused by the disaster will be over.

